# The Influence of Heat and Mechanical Treatment of Concrete Rubble on the Properties of Recycled Aggregate Concrete

**DOI:** 10.3390/ma12030367

**Published:** 2019-01-24

**Authors:** Edyta Pawluczuk, Katarzyna Kalinowska-Wichrowska, Michał Bołtryk, José Ramón Jiménez, José María Fernández

**Affiliations:** 1 Faculty of Civil and Environmental Engineering, Bialystok University of Technology, 15-351 Białystok, Poland; e.pawluczuk@pb.edu.pl (E.P.); k.kalinowska@pb.edu.pl (K.K.-W.); m.boltryk@pb.edu.pl (M.B.); 2 School of Engineering Sciences of Belmez, University of Córdoba, 14240 Belmez, Spain; um1feroj@uco.es

**Keywords:** recycled concrete aggregate, recycled aggregate concrete, concrete gravel, thermo-mechanical treatment, microstructure, X-ray diffraction

## Abstract

Concrete is a building material commonly used for ages. Therefore, in the result of repairs or demolition of building structures, large amounts of concrete rubble are created, which requires appropriate management. The aim of the realized research was to determine the influence of heat and mechanical treatment of concrete rubble on the properties of recycled aggregate concrete. The research experiment included 12 series, with three variables: X_1_—roasting temperature (300, 600, 900 °C), X_2_—time of mechanical treatment (5, 10, 15 min), X_3_—content of coarse recycled aggregates (20, 40, 60% by volume). Two additional series containing recycled aggregate without treatment and natural aggregate were also prepared. Established properties of individual aggregates have confirmed a positive effect of thermo-mechanical treatment. Then, based on the results of compressive strength, flexural strength, Young’s modulus, volumetric density, water absorption, water permeability and capillarity, the most favourable parameters of heat and mechanical treatment of concrete were determined. The test results showed that appropriate treatment of concrete rubble allows to obtain high-quality coarse aggregate and valuable fine fraction. This was also confirmed by the macro- and microscopic observations of the aggregate and separated cement paste. Works realized on the concrete recycling method resulted in obtaining a patent PAT.229887.

## 1. Introduction

Concrete is the single most widely used construction material in the world but also one of the most environmentally unfriendly. Its manufacture consumes a large amount of non-renewable natural resources (aggregates: 80%), Portland cement (10%), supplementary cementitious materials (3%), and water (7%). Its production is responsible for 5% of anthropogenic worldwide CO_2_ emissions [1].

Natural aggregates (NAs) used in the manufacture of concrete are inert granular materials such as sand, gravel or crushed stone. Natural gravel and sand are usually dug or dredged from a pit, river, lake or seabed. Crushed aggregate is produced by crushing quarry rock, boulders or large-size gravel. Currently, the global aggregate production is estimated at 40 billion tons, which is leading to a depletion of natural resources, high energy consumption, and impacts on the environment [2]. 

Countries that are consuming the largest amounts of aggregates are China (38%), India (13%), other Asian countries (12%), Africa (8%), USA (6%), EU + EFTA (8%), C&S America (6%) and Oceania (4%). Approximately 58% of aggregates are used for the manufacture of concrete and mortar. The use of recycled aggregates from construction and demolition waste (CDW) in the manufacture of concrete and mortar is a viable alternative to reduce the unsustainable level of consumption of natural aggregates worldwide and avoid landfilling CDW [3,4].

Concrete structures are demolished at the end of their service life generating concrete waste which can be recycled in treatment plants [5]. In these plants, concrete rubble is crushed to reduce the grain size in order to produce recycled concrete aggregates (RCAs). The RCAs can be screened to obtain two fractions: coarse recycled concrete aggregates (CRCAs) and fine recycled concrete aggregates (FRCAs). Most of the RCA studies have been focused on the use of CRCAs, because of the worse physico-mechanical and chemical properties of FRCAs. Hence, most technical recommendations (e.g., codes, instructions) exclusively propose the use of CRCAs for RCA production [4].

Recycled concrete aggregates are composed of natural aggregates covered with a porous layer of cement paste. The recycled aggregate’s quality is closely related to the adhered cement paste properties, since the bond between the primitive natural aggregates and the cement paste is usually weak in the interfacial transition zone [6]. It is widely accepted that the presence of cement paste in RCA justifies its worse physico-mechanical and chemical properties compared to NAs. Recycled concrete aggregate texture is more angular, rough, and porous than NAs [7]. Recycled concrete aggregate density is lower than NAs and the absorption of water is greater. Recycled concrete aggregates have a lower resistance to fragmentation than NAs due to the adhered cement paste [8]. From a chemical point of view, RCAs contain more sulphates and soluble salts than natural aggregates [9,10]. 

The replacement of NAs by RCAs reduces the new concrete’s mechanical and durability properties. Nevertheless, the use of high-quality RCAs may result in concrete with similar compressive strength, flexural strength, Young’s modulus and shrinkage than conventional concrete, even with high replacement ratios. The cement paste content is the main factor influencing creep deformation, which can limit the use of RCA in structural concrete application. The conventional concrete shrinkage prediction models are also affected by the cement paste adhered to the RCA, since it modifies the internal curing conditions, especially when the aggregates are used pre-saturated. From a durability point of view, the use of RCA increases porosity, water absorption, carbonation depth, chloride ion penetration, and decreases resistance to freezing-thawing, sulphate attack or resistance to abrasion [11,12].

In order to improve RCA quality, many researchers have applied different techniques for removing the adhered cement paste from the original natural aggregates. The main methods used so far are: (i) acid treatment, in which the RCA are soaked in acidic solutions (nitric acid, hydrochloric acid, sulphuric acid, phosphoric acid, acetic acid) [13,14,15,16], (ii) thermal treatment in which the RCA is treated thermally in a furnace and immediately immersed in water [8,15], (iii) mechanical treatment in which the RCA is treated in a jaw crusher or ball mill [15]. These methods improve the physico-mechanical and chemical properties of RCA. The use of treated RCA significantly improves the compressive strength of concrete compared to that of untreated recycled aggregates. Pandurangan et al. [15] recommend the mechanical treatment against the use of acids because it is faster, economical and eco-friendly, although acid treatment methods are more effective in improving the bond strength of reinforcement and concrete.

However, studies on the combined effect of different treatment methods are scarce. This study analyses the combined effect of thermal and mechanical treatment on RCA properties, as well as the mechanical and durability properties of the new concrete. For this purpose, three variables have been tested: roasting temperature (300, 600, 900 °C), time of mechanical treatment (5, 10, 15 min) and content of coarse RCA in concrete mixtures. The best option to manufacture structural concrete with treated RCA is selected. From a practical point of view, the results of this work are especially interesting in emerging countries with a shortage of natural aggregates and where the production of high-quality RCAs is a necessity.

## 2. Materials and Methods 

### 2.1. Materials

The Portland cement CEM I 42.5R, from the Ożarów cement plant (Poland) meeting the requirements of PN-EN 197-1 standard was used for the test samples. The following natural aggregate was used for the concrete mix: sand, fraction 0-2 mm; gravel, fraction 2-4 mm; granite, fractions 4–8 and 8–16 mm. The recycled aggregate was obtained from crushing of concrete based on granite aggregate. After crushing, the debris was subjected to heat and mechanical treatment and then screened into 4–8 mm and 8–16 mm fractions. A superplasticizer based on modified polycarboxylates was used.

### 2.2. Methods

The aggregate bulk density test was carried out in according to EN 1097-3. The density of the concrete mixture was determined according to EN 12350-6 and the consistency according to EN 12350-2. The compressive strength of concrete was tested on cubic samples of 150 mm × 150 mm × 150 mm according to EN 12390-3. Determination of modulus of elasticity was carried out on cylindrical samples of 150 mm × 300 mm based on EN 12390-13. The concrete flexural strength test was carried out on samples of 100 mm × 100 mm × 500 mm according to EN 12390-5. The concrete density and water permeability were determined on cubic samples of 100 mm × 100 mm × 100 mm according to EN 12390-7 and EN 12390-8, respectively. The concrete water absorbability test was carried out according to the Polish Standard PN-88/B-06259.

## 3. Technology of Recycled Aggregate Production

### 3.1. The Origin of Concrete Rubble

The recycled aggregate was obtained from processing of 3-year old laboratory samples of the dimensions of 150 mm × 150 mm × 150 mm and 150 mm × 150 mm × 750 mm, taken as control samples during the construction of concrete pavements. The composition of the concrete mixture of these samples is presented in Table 1. The designed concrete class was C35/45.

### 3.2. The Preliminary Crushing of the Samples

The preliminary crushing of the samples due to their excessive dimensions exceeding the inlet of the laboratory crusher took place during the concrete compressive and flexural strength test. The results of those tests are presented in Table 2.

### 3.3. Primary Crushing of Concrete Rubble

**Figure 1:**
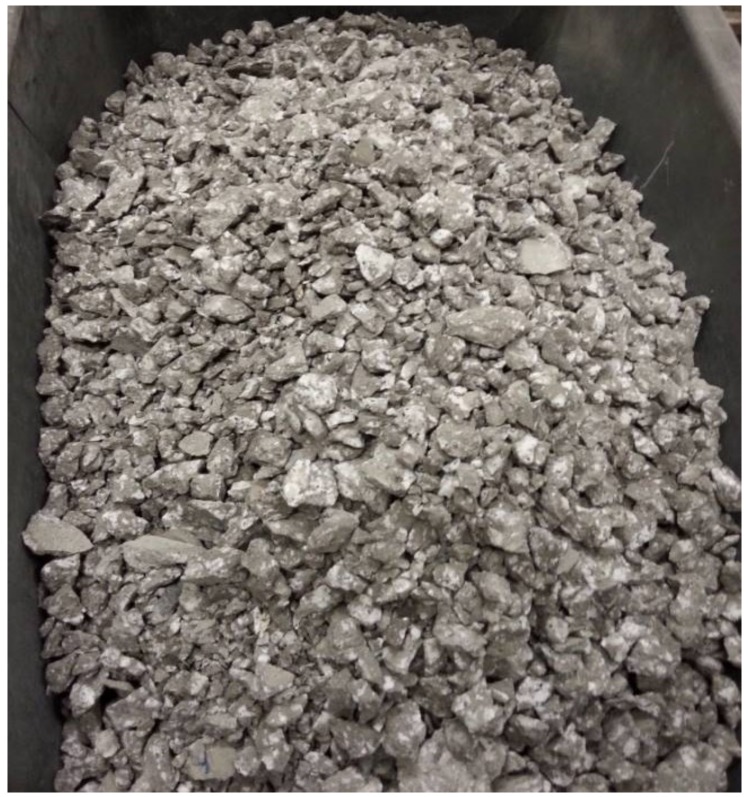
Coarse recycled concrete aggregate.

The basic crushing of previously disintegrated concrete samples was carried out in the jaw crusher. The concrete was crushed to the grain size d ≤ 20 mm and then it was sieved in order to separate fine and dust fractions (<4 mm). The obtained coarse aggregate is shown in Figure 1.

### 3.4. The Thermal Treatment of Recycled Concrete Aggregate

The recycled aggregate of 4–20 mm fraction was then roasted in a CT 100 EK ceramic laboratory furnace at a rated temperature of 1250 °C. The recycled aggregate calcination is necessary in order to dehydrate the cement paste, which reduces adhesion of the hardened mortar to the aggregate grains and finally makes it easier to remove it from the surface of the aggregate.

**Figure 2:**
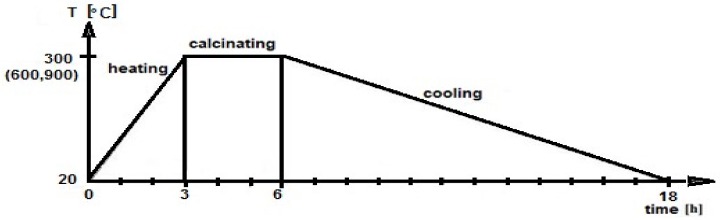
The heating process of recycled concrete aggregate.

The heat treatment time was set at 3 h. According to the plan of the experiment, the roasting temperature was set at 3 levels: 300 °C, 600 °C, and 900 °C. The heating process of recycled concrete aggregate is shown in Figure 2.

After thermal treatment and cooling, the recycled aggregate was mechanically processed. 

### 3.5. The Mechanical Treatment of Recycled Concrete Aggregate

The last stage of the recycled aggregate quality improvement was mechanical processing. The Los Angeles drum was used for that purpose. The machining time was set at 5 min, 10 min, and 15 min, respectively. It allowed for the final separation of the cement mortar from the surface of coarse aggregate grains. Then the treated material was screened in order to separate fractions of 4–8 mm and 8–16 mm required for further tests.

### 3.6. Impact of Thermo-Mechanical Treatment on the Amount of Separated Cement Mortar

After heat and mechanical treatment the percentage content of separated cement mortar (<4 mm) in relation to the initial mass of recycled aggregate was determined. The results are presented in Table 3.

Mortar obtained, after grinding, can be used as an active addition to cement composites or as a substitute for part of the binder [17,18,19,20]. It can also be a component of lime–sand products. The test results will be described in another paper.

After the analysis of the results it was observed that the abrasiveness of the aggregate mortar increased gradually with temperature and time of heat and mechanical treatment until the moment of roasting the aggregate in 900 °C, where sudden and unexpected increase in abrasiveness occurred. After the mechanical treatment of the aggregate roasted at 900 °C, almost the entire series was sieved through a 4-mm screen. It turned out that roasting at such a temperature destroyed the granite structure. To confirm this, an additional previously unplanned experiment was carried out, roasting the natural granite at 900 °C for 3 h. It turned out that the granite lost its properties at 900 °C, as the aggregate was destroyed in the moment of light pressure. A significant decrease in the mechanical properties of granite and a sudden increase in its porosity due to high temperatures above 800 °C was also observed by other authors [21,22]. 

## 4. Preparation of a Research Plan

### 4.1. Selection of Variables and Development of the Experiment Plan

An experiment based on three variables was planned in order to investigate the influence of the improvement of the quality of coarse recycled aggregate on selected concrete properties: X_1_—temperature of recycled aggregate heat treatment; X_2_—mechanical treatment time; and X_3_—percentage amount of coarse recycled aggregate. The levels of their variability are shown in detail in Table 4.

The theory of statistical planning of experiments was used to determine the research plan. The experiment and calculations were carried out in accordance with the statistically determined, sequential Hartley’s PS/DS-P:Ha3 plan, built on hypercube. The Hartley’s DoE (Design of the Experiment) enables to check repeatability of results, to find which inputs and their interactions influence the output significantly, to calculate regression equation and check its adequacy with test results.

Upon the basis of abovementioned variables, an experimental plan including 12 research series and 2 control series was established. For the first control series (13), the recycled concrete aggregates were used directly after crushing, without heat and mechanical treatment (RCAwt), while the next control series (14) was made with granite (NA). Table 5 shows the detailed experimental plan including the real and coded values of the variables.

### 4.2. The Composition of the Concrete Mixtures

Table 6 shows the composition of the concrete mix depending on the percentage of recycled aggregate. The compositions of concrete mixes were designed on the assumption of a constant amount of CEM I 42.5R cement in the amount of 400 kg/m^3^ and a constant w/c ratio.

## 5. Test Results and Discussion

### 5.1. Properties of Used Aggregates

Table 7 presents the test results of selected properties of recycled and natural aggregates in the range of bulk density in a dry and saturated state and water absorption.

The above results indicate that recycled aggregates roasted at a temperature of 600 °C were characterized by bulk density which was closest to natural aggregate, both in dry and saturated state. Also the water absorption of this aggregate was the lowest among the tested recycled aggregates (3.11% and 3.70%). This was mainly due to the effective removal of the old cement mortar from the aggregate grains, the presence of which jeopardizes the RCA properties, in particular the increase in its water absorption. It was due because at the temperature of about 600 °C, calcium hydroxide mainly located in the contact zone between the aggregate grain and the cement paste was dehydrated, making it easier to remove the mortar during mechanical treatments. The absorbability of untreated aggregates was much higher and amounted to 6.85% and 7.83%, respectively. Slightly worse results were obtained in case of aggregates roasted in 300 °C. The results indicated that recycled aggregate not subjected to the heat and mechanical treatment was characterized by definitely the lowest bulk density, which was caused by its developed surface due to the presence of cement mortar and irregular shape hindering tight packing of grains in the container, which is in agreement with most of the studies on physical properties of recycled aggregates [7,9]. The presence of cement mortar on the surface is also confirmed by increasing bulk density in the saturated state by about 18% in compare to the dried state. Roasting of granite in 900 °C caused damage to the grains structure, the breakdown of which into smaller fragments resulted in an increase of the bulk density in relation to the aggregate not roasted by better packing of particles in the vessel. However, this material was still characterized by relatively high water absorption, about five to six times higher in comparison to the natural aggregate.

### 5.2. Properties of Concrete Mixture

The results of the consistency of concrete mixtures measured by the slump test and their density are presented in Table 8.

Upon the basis of the analysis of the above results, it can be seen that the highest slump of 225 mm cone corresponding to the consistence class S5 was recorded for 14 control series. The lowest slump of 140 mm cone corresponding to the consistence class S3 was recorded for series 13. The reason was the use of recycling aggregate without the heat and mechanical treatment having the highest amount of cement mortar absorbing water.

For the samples from series 5 and 9 containing 60% of recycled aggregate roasted in 900 °C, the consistence class was not determined, as no slump was observed here. In other series, the consistence values were similar to each other and corresponded to class S4.

### 5.3. Properties of Hardened Concrete 

Table 9 shows the average results of concrete properties tests for the individual series of the experiment, together with the control series. Compressive strength (f_cm,28_), flexural strength (f_fm,28_), Young’s modulus (E), volume density (D), water absorption (WA), water permeability (WP) as water depth penetration and water capillarity as increase of sample mass (WC) have been determined.

The test results were statistically analyzed in order to determine an approximating function describing changes in selected physico-mechanical properties of the concretes. The analyses included variance, a calculation of regression coefficients and an assessment of the regression coefficients’ significance. The function describing changes in the physico-mechanical properties of concretes adopted a form of the second degree polynomial (1):

Y = b_0_ + b_1_x_1_ + b_2_x_2_ + b_3_x_3_ + b_4_x_1_x_2_ + b_5_x_1_x_3_ + b_6_x_2_x_3_ + b_7_x_1_^2^ + b_8_x_2_^2^ + b_9_x_3_^2^ (1)

Calculations were performed using Statistica Version 13. The equations for the concrete’s compressive strength, flexural strength, Young’s modules (E), volume density, water absorption, water permeability and water capillarity which considers only significant regression coefficients at α = 0.05, are presented in Table 10.

The statistical analysis revealed that all analysed physical and mechanical properties of concrete were significantly affected by the roasting temperature and recycled aggregate content. On the other hand, the time of mechanical treatment usually did not significantly affect the obtained values of parameters, therefore the variable x_2_ was assumed at the average level (x_2_ = 0, 10 min). Due to the incomplete research plan the test result analysis was prepared upon the basis of established regression equations.

#### 5.3.1. Compressive Strength

**Figure 3:**
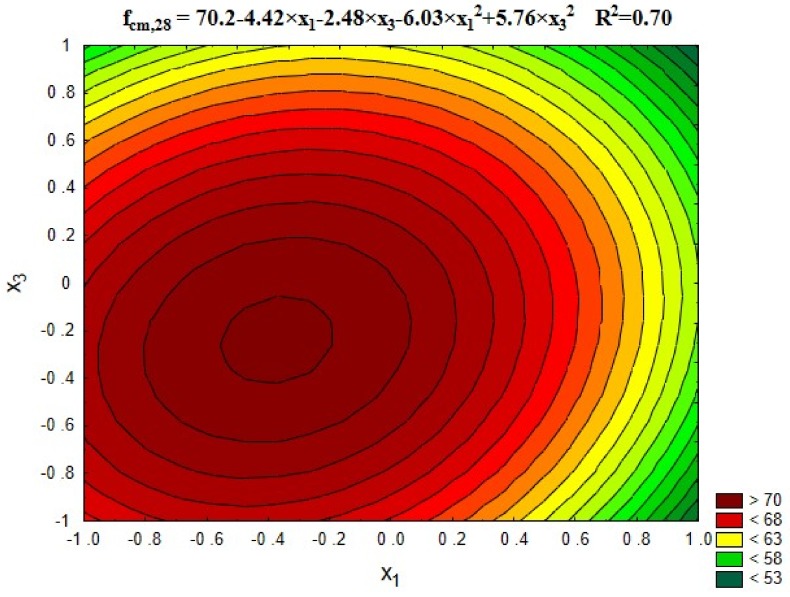
The changes in compressive strength of concrete, depending on x_1_ and x_3_ (x_2_ = 0).

**Figure 4:**
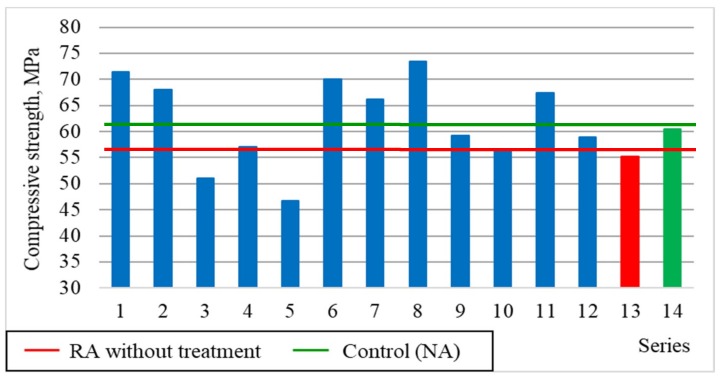
The compressive strength of concretes compared with series 13 and 14.

Figure 3 shows changes in concrete compressive strength depending on the roasting temperature of rubble (x_1_) and recycled aggregate content (x_3_). Figure 4 shows the average strength results obtained for the individual research series in comparison with the series containing the untreated recycled aggregate (13) and the control series containing the natural aggregate (14).

Upon the basis of Figure 3, it can be stated that the heat treatment of recycled aggregates positively influenced the concrete compressive strength, in particular in the temperature range from 300 °C to 600 °C. At about 600 °C, the disintegration of portlandite into lime and water occurred, which simplified the removing of the cement mortar from the surface of coarse recycled aggregate. After detachment, some of the lime remained on the surface of the aggregate and then reacted with the new cement paste. This fact caused a better sealing of the contact zone which resulted in the increase of the mechanical properties.

In higher temperatures (900 °C), the aggregate structure was damaged, resulting in a sudden decrease in compression strength by about 17% on the average. It is also confirmed by the amount of cement mortar separated from the aggregate surface after its heat and mechanical treatment (Table 2). As shown in Figure 4, in the presence of 60% of recycled aggregate subjected to treatment, the results higher by approx. 12% on the average were obtained for the concrete compressive strength than for untreated aggregate (series 13) and a few percent higher in comparison with natural aggregate (series 14). The use of recycled aggregate without the heat and mechanical treatment decreased the compressive strength of concrete by three classes compared to the control concrete.

#### 5.3.2. Flexural Strength

**Figure 5:**
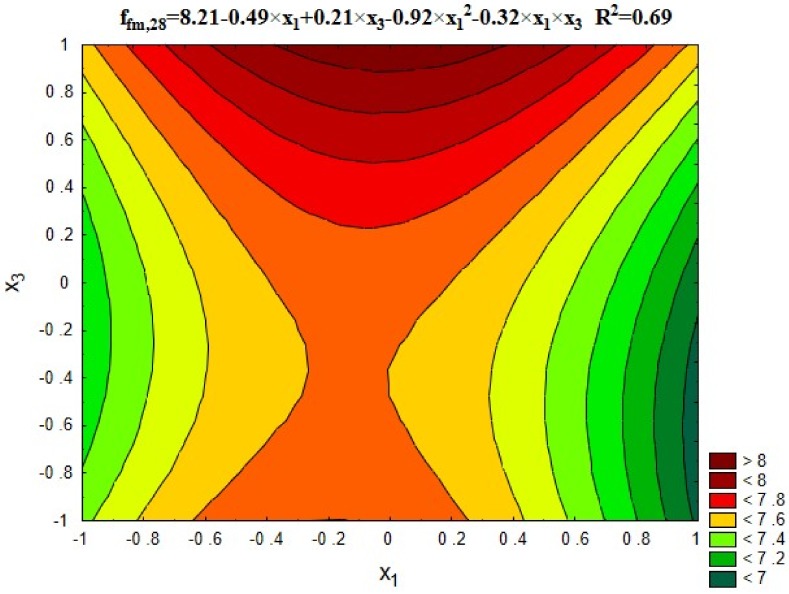
Changes in flexural strength of concrete depending on x_1_ and x_3_ (x_2_ = 0).

**Figure 6:**
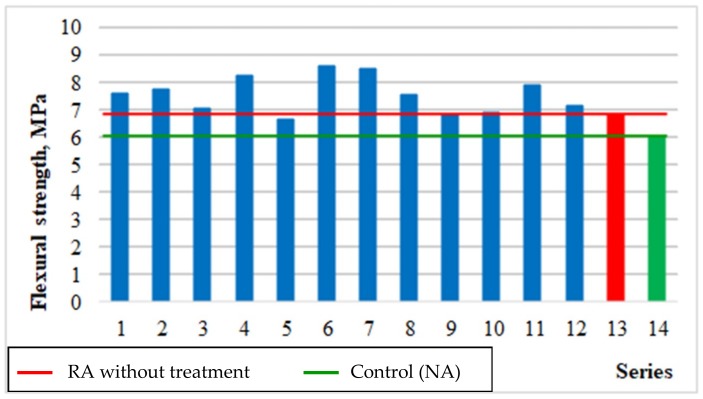
The flexural strength of concretes compared with series 13 and 14.

Figure 5 and Figure 6 show changes in the concrete flexural strength depending on the roasting temperature of rubble (x_1_) and recycled aggregate content (x_3_), respectively, as well as the average flexural strength results obtained for the individual test series compared to the series with untreated aggregate (13), and the control series with the natural aggregate (14).

Figure 5 reveals that concretes with recycled aggregate treated in 600 °C obtained the most favourable bending strength. This is the optimal temperature due to decomposition process of calcium hydroxide (portlandite) located in the contact zone between the aggregate and cement paste. As a result, the removal of cement mortar from the grain surface is facilitated, which is confirmed by the results presented in Table 3. However, traces of mortar remain on the aggregate grains surface, which forms a more permanent bonds with new cement paste. This results in higher flexural strength for all test series than for the control series (14). It should also be noted that in all series the flexural strength of concrete ranged from 10% to 14% of their compressive strength.

#### 5.3.3. Young’s Modulus E

**Figure 7:**
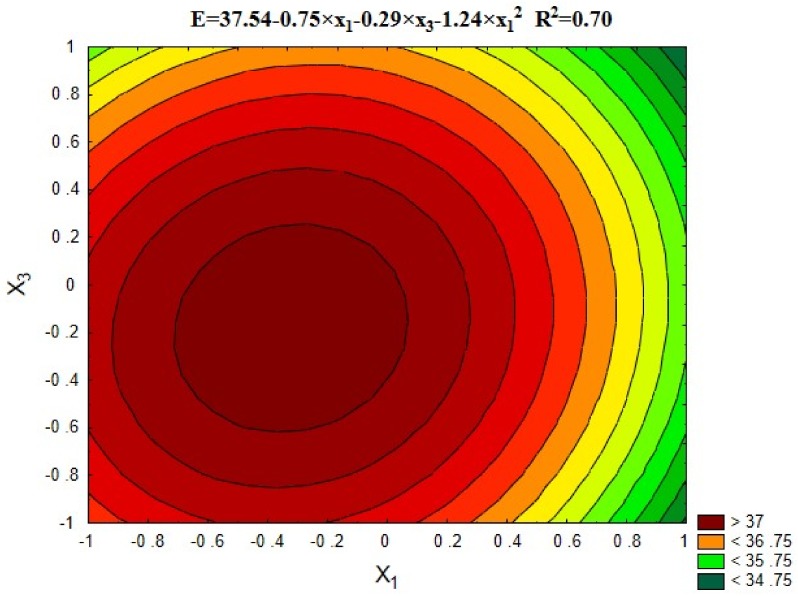
The changes in Young’s Modulus depending on x_1_ and x_3_ (x_2_ = 0).

**Figure 8:**
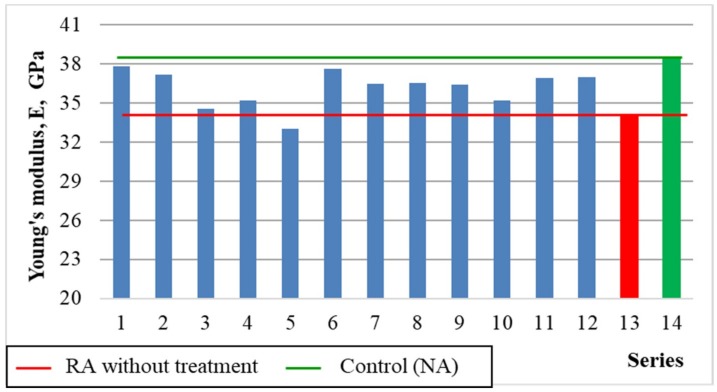
Young’s Modulus of concretes compared with series 13 and 14.

Figure 7 and Figure 8 show the results of Young’s Modulus (E) depending on variables x_1_ and x_3_, as well as the average results compared to the series with untreated recycled aggregate (13), and the control series with natural aggregate (14).

**Figure 9:**
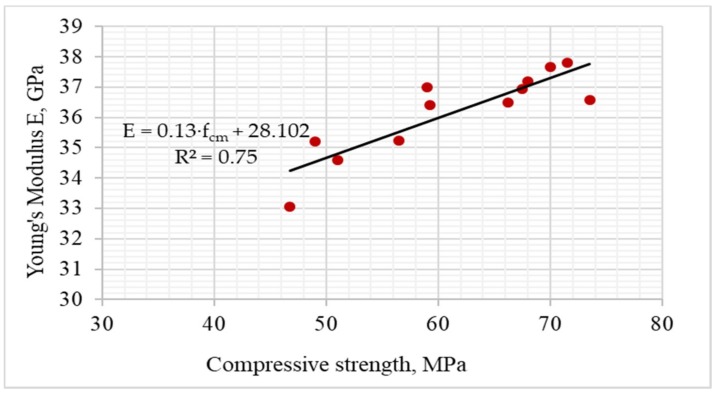
Relationship between the Young’s Modulus for the individual series and the compressive strength of concrete.

**Figure 10:**
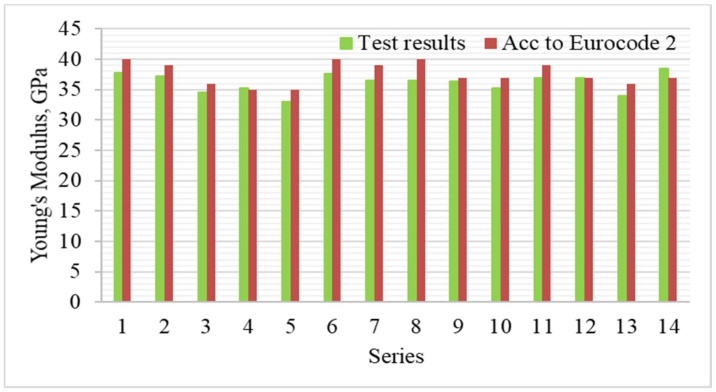
The comparison of the results of Young’s Modulus with standard results according to EN 1992-1-1 [23].

The important factors influencing the strength and deformation properties of concrete are the adhesion between cement paste and aggregate grains, and the microstructure of the transition zone in the area of stress concentration due to the difference in modulus of elasticity values of the hardened paste and the aggregate. Figure 7 reveals that the highest modulus of elasticity was observed for series with the aggregate roasted up to 600 °C. The process of roasting and mechanical treatment resulted in removing the old cement mortar from the aggregate surface, which improved the adhesion of the aggregate to the cement paste. In such way the modulus of elasticity exceeding 35 GPa was obtained. The exception are series with aggregates roasted in 900 °C, which caused damage to the aggregate structure and significant deterioration of concrete properties. Figure 9 shows the relationship between the modulus of elasticity for the individual series and the compressive strength of concrete, while Figure 10 shows a comparison of the results with standard values (for quartzite aggregates, according to EN 1992-1-1 [23]).

Figure 9 shows that there is correlation in the experiment between the compressive strength of concrete and Young’s Modulus. The highest value of Young’s Modulus was obtained in the control series (14) with granite aggregate, which even exceeded the standard value. In most series, the modulus of elasticity was lower when comparing to determine the standard by about 4% on the average. Similar results were obtained by other authors, where the addition of 100% of recycled high-quality aggregate resulted in reduction of Young’s Modulus by only 11% [24]. However, it can be seen that changes in modulus of elasticity values in the individual series due to heat and mechanical treatment and recycled aggregate content were not significant. In most series, the E value decreased by 2–10% comparing to the control series. In series with recycled aggregate roasted at 900 °C the decrease was even 14%. This is confirmed by other authors, which obtained much higher drops in Young’s Modulus values with an increase of recycled coarse aggregate content [25,26].

#### 5.3.4. Volume Density

**Figure 11:**
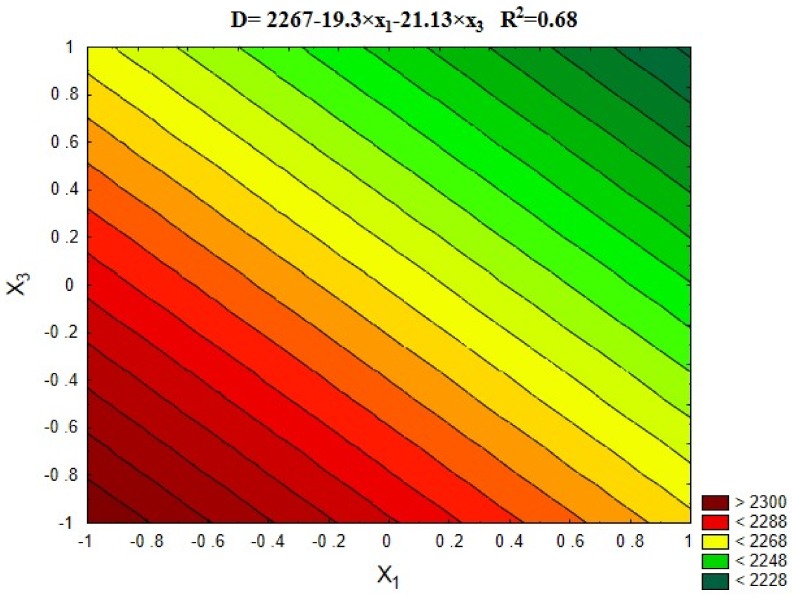
The changes in volume density of concrete depending on x_1_ and x_3_ (x_2_ = 0).

**Figure 12:**
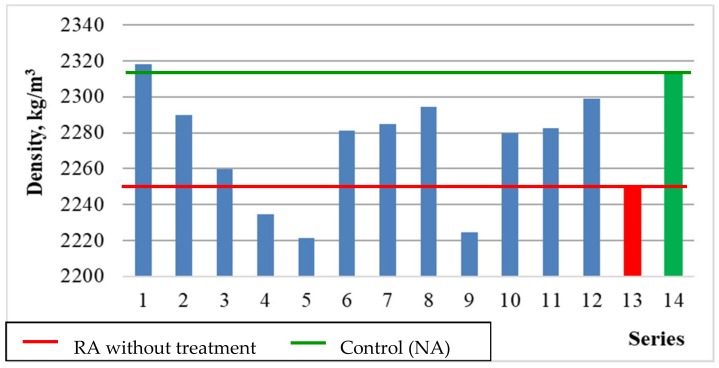
The volume density of concretes compared with series 13 and 14.

Figure 11 and Figure 12 show the results of the concrete volume density depending on the variables x_1_ and x_3_, and the average results comparing to the series with untreated RA (13) and the control series with natural aggregate (14).

From Figure 11 and Figure 12 it results that both the roasting temperature up to 600 °C and the recycled aggregate content influenced the concrete density. In general, the concrete with the natural aggregate was characterized by the highest density, which results from the highest density of granite present in it. The increase of recycled aggregate content resulted in decrease of the concrete density. It results from the content of cement mortar present on the aggregate surface. However, it can be noticed that the differences in concrete density throughout the experiment are small and reach the order of 4%. This is due to the good quality of the rubble obtained from high-quality source concrete (C45/55).

#### 5.3.5. Water Absorption

**Figure 13:**
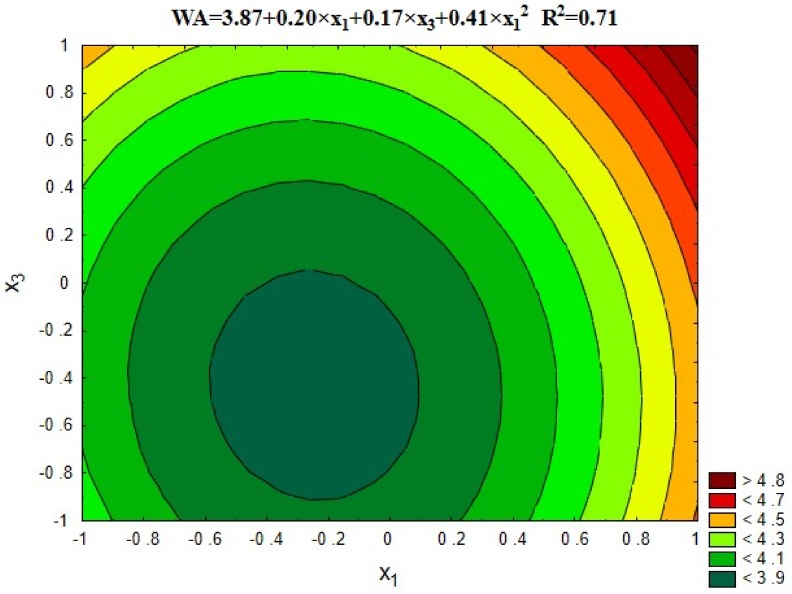
The water absorption of concrete depending on x_1_ and x_3_ (x_2_ = 0).

**Figure 14:**
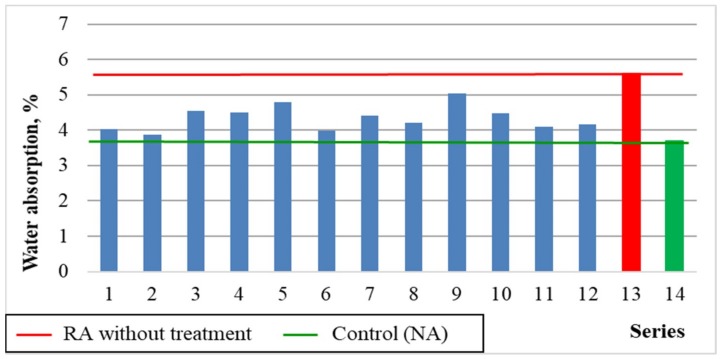
The water absorption of concretes compared with series 13 and 14.

The results of concrete water absorption for each series of the experiment are shown in Figure 13. Figure 14 shows a comparison of average results with the series with untreated recycled aggregate (13) and the control series with natural aggregate (14).

The best concrete water absorption was observed in the case of using 20–40% of recycled aggregate roasted in temperatures up to 600 °C. In such cases, the absorbability approaches the value for the control concrete (≤4% for series 2 and 6). It results from effective removal of porous cement mortar from the recycled aggregate surface. Its high absorbability causes many problems connected with determining the amount of water added to the recycled concrete aggregate in order to obtain the required consistency. In series 13 with untreated aggregate, the absorbability of concrete has increased significantly by 66%, compared to control concrete. Clearly higher absorbability of recycled aggregate concrete compared to the natural aggregate concrete was also observed by other researchers [27,28]. Removing the cement mortar from the aggregate surface as a result of proposed treatment facilitates the design and execution of the concrete mix and significantly improves the properties of recycled aggregate concrete, bringing it closer to the properties of control concrete.

#### 5.3.6. Water Permeability (Depth of Penetration)

**Figure 15:**
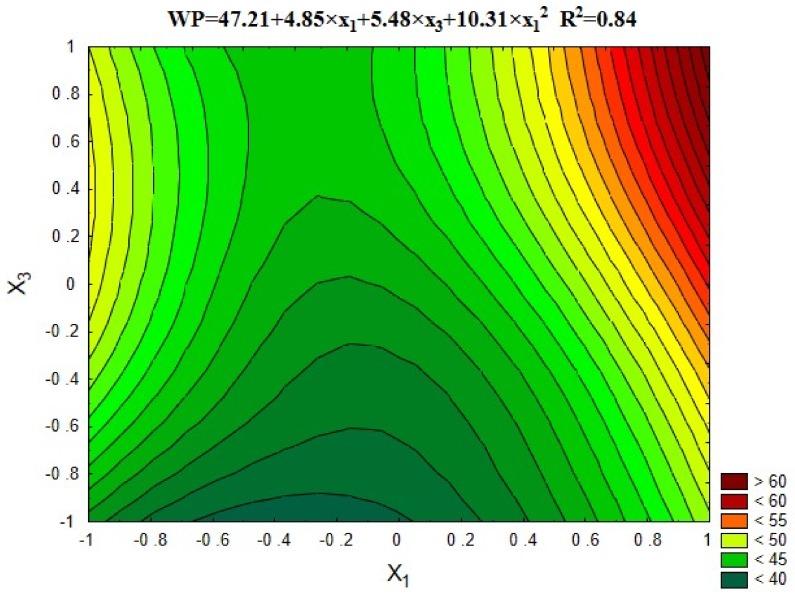
The changes in the depth of water penetration depending on x_1_ and x_3_ (x_2_ = 0).

**Figure 16:**
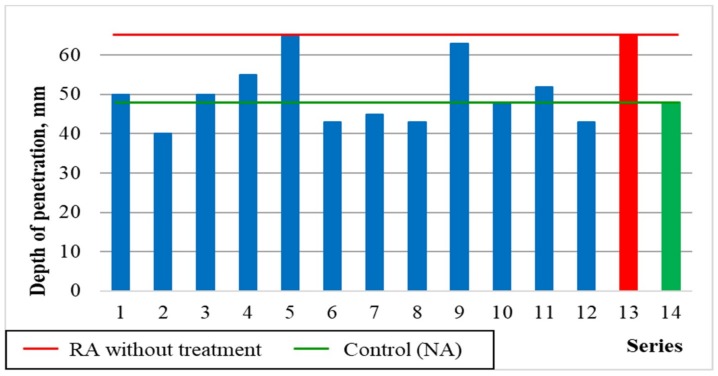
The water penetration of concretes compared with series 13 and 14.

Figure 15 and Figure 16 show the results of water permeability for concrete, expressed by the depth of water penetration under pressure, depending on the variables x_1_ and x_3_, as well as the average results in comparison with the series with untreated aggregate (13) and with the control series with natural aggregate (14).

As shown in Figure 15, the penetration depth of pressurized water increases with the increase in recycled aggregate content, which is related to the presence of porous cement mortar on the grain surface. It can also be seen that the heat and mechanical treatment of recycled aggregate up to 600 °C had a positive effect on the capillary structure of concrete, reducing the depth of water penetration in comparison with the control concrete by 6–10%. It may be assumed that the highest water penetration depth was obtained for the series with untreated recycling aggregate (series 13) due to the highest content of porous cement mortar significantly improving the concrete permeability (Figure 16). Similarly, unfavourable results were also obtained for series containing 60% of recycled aggregate roasted in 900 °C. As previously stated, such treatment damaged the grain structure of the aggregate, leading to the increase of its porosity and water permeability. Moreover, it should be noted that in none of the tested series the depth of water penetration exceeded 65 mm, which proves the relatively compact structure of prepared samples.

#### 5.3.7. Water Capillarity

**Figure 17:**
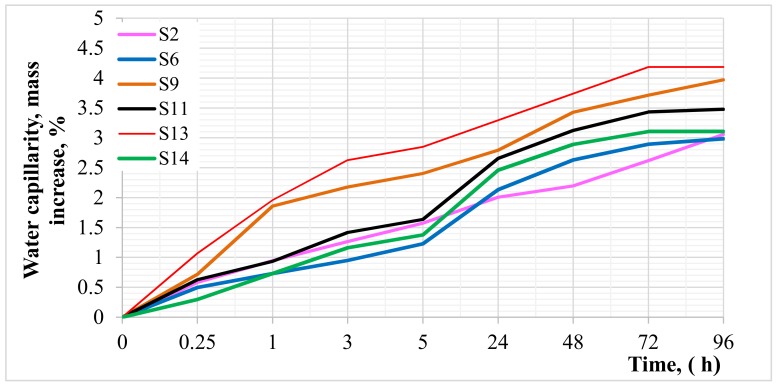
Average values of water capillarity as percent mass increase for selected series.

Figure 17 shows the course of capillary water rising in selected test series, expressed by the percentage mass of water absorbed by the concrete in time.

Figure 17 shows that concrete with untreated aggregate and aggregate roasted at 900 °C showed the highest capillary water pull capacity. It confirms the results obtained in case of water permeability. It should be noted that the process of water extraction in those series was significantly more intensive, especially during the first hours of the test, which was influenced by the presence of a porous cement mortar. The results of research by other authors also confirmed a significant increase in capillary water rising in case of recycled aggregates compared to the natural aggregate [28,29]. In other series, the course of the test was similar to the results obtained for control concrete, which confirms that the cement mortar was almost completely removed from the aggregate surface as a result of heat and mechanical treatment.

## 6. Structure and Microstructure Tests

### 6.1. Optical and Scanning Microscopy

**Figure 18:**
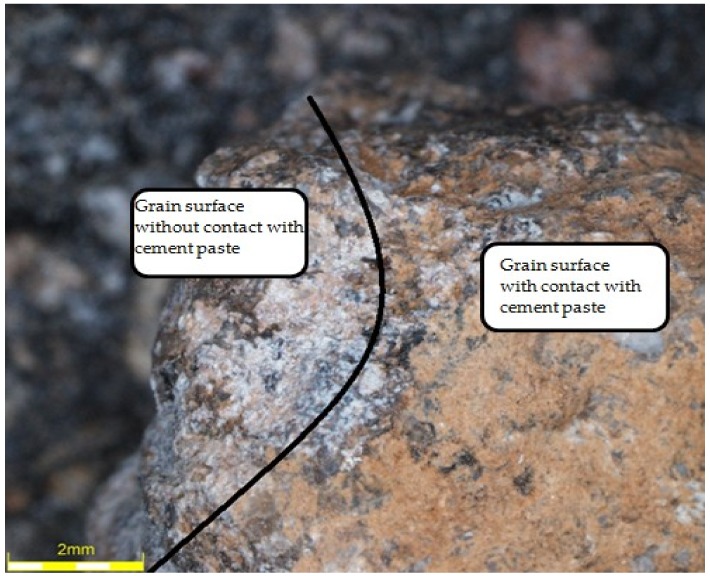
The granite after thermo-mechanical treatment.

**Figure 19:**
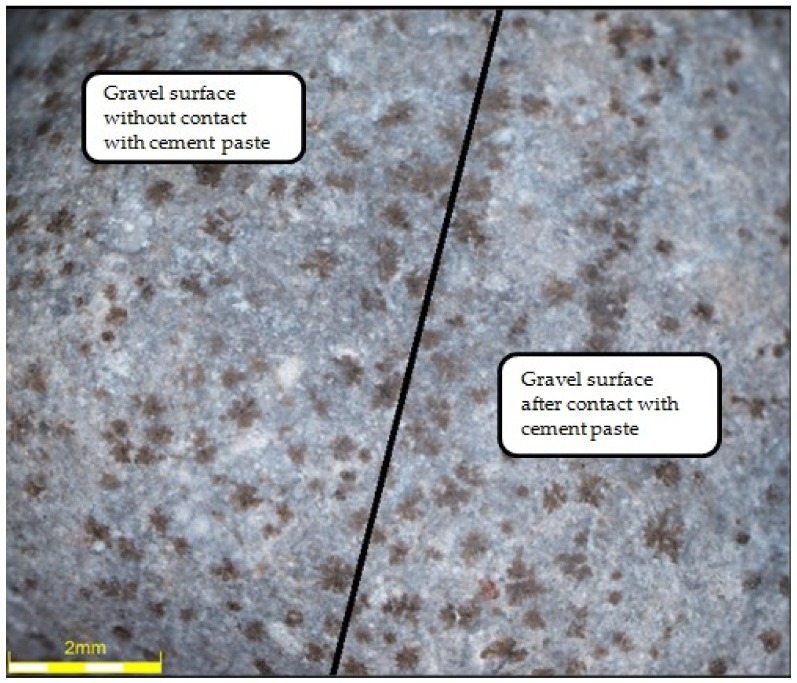
Gravel after thermo-mechanical treatment.

The observation of aggregates using an optical microscope was aimed for comparing changes in their surface as a result of contact with cement slurry during the hydration process, as well as after the applied heat and mechanical treatment. For this purpose, the cylindrical samples of dimensions of 50 mm × 48 mm from cement paste (CEM I 42.5R and water) of w/c = 0.45 were made, in which the natural aggregates (granite and gravel, respectively) were immersed. Previously part of the grain was covered with stearin in order to prevent its direct contact with the paste. Samples were kept in water for 3 months before testing. After drying the samples were subjected to the heat and mechanical treatment (i.e., roasting for 60 min in 650 °C and grinding in the Los Angeles drum). Then the obtained aggregates were observed under an optical microscope in order to determine the possible damage or visible changes in the external structure. Figure 18 and Figure 19 show the granite aggregate and gravel, respectively, subjected to the heat and mechanical treatment.

Figure 18 and Figure 19 show that the removal of cement slurry from the granite aggregate as a result of the applied heat and mechanical treatment was not as effective as in case of gravel aggregate. A part of the slurry remained on the surface of the granite grain, colouring itself light brown. The difference between the part of the grain surface that was in contact with the cement slurry and another part is quite obvious (Figure 18). On the other hand, in the case of the gravel aggregate, the difference is not noticeable, which proves the beneficial effect of heat and mechanical treatment on this type of aggregate. However, the surface of the gravel contacting the slurry was locally developed due to the adjacent slurry, which has also become yellowish. In addition, the mechanical treatment made the previously smooth aggregate surface uneven and rough, which makes it easier for the grain to combine with cement paste in the new cement composite. 

**Figure 20:**
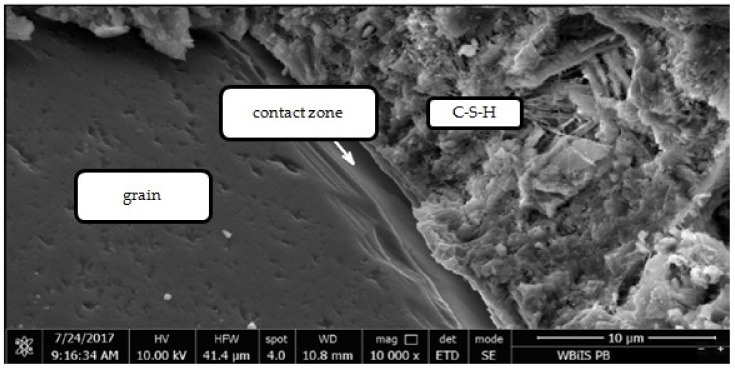
Contact zone between natural gravel aggregate and cement paste.

**Figure 21:**
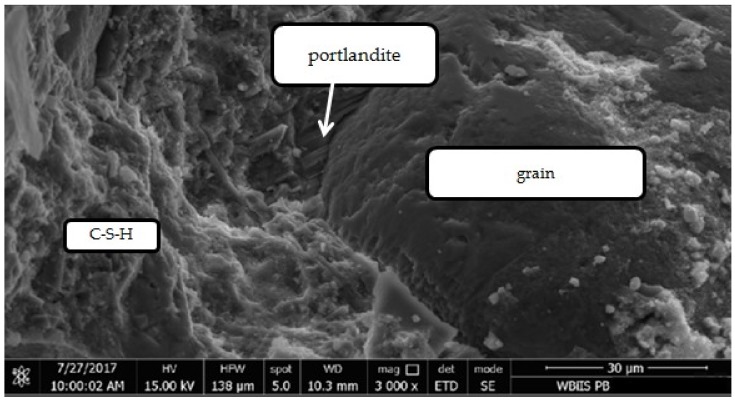
Contact zone between recycled aggregate without thermo-mechanical treatment and cement paste.

**Figure 22:**
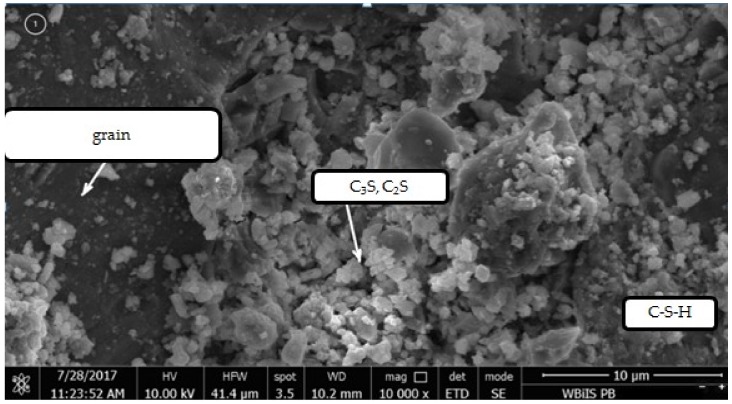
Recycled aggregate after thermo-mechanical treatment.

**Figure 23:**
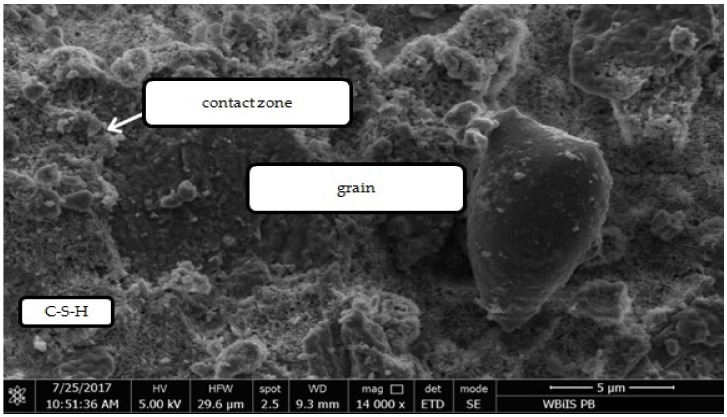
Contact zone between recycled aggregate after thermo-mechanical treatment and cement paste.

In the case of the granite aggregate, the presence of grout on its surface does not significantly change the grain shape. According to other researchers, the granite aggregate is characterized by higher chemical activity in contact with cement paste than the gravel. They have also found that the microstructure of the cement paste surrounding the gravel grain is loose and porous due to higher absorbability of the aggregate. The microstructure of the paste around the granite is denser than around the gravel, small pores and defects are visible and many groups of hydrates are closely bonded with each other. It is therefore difficult to identify the paste and the aggregate [30]. The physical interaction between the aggregate and the cement slurry depends mainly on the roughness of the surface and the absorbability of the aggregate. Due to the fact that granite has a compact and rough structure with low absorbability and surface chemical activity, the adhesion of the paste to its surface is much better than in case of the gravel aggregate. The smooth surface of the gravel, at which the portlandite tiles are located, facilitates the separation of the cement paste through the proposed treatment. Therefore, as a result of heat and mechanical treatment more paste residues were observed on the surface of granite grains than on the gravel grains. Figure 20, Figure 21, Figure 22 and Figure 23 show the observations made with use of scanning microscopy for the contact zone in the control concrete with gravel aggregate and in the concrete with recycled aggregate not roasted properly and subjected to the heat and mechanical treatment.

Figure 20 shows the contact zone typical for the ordinary concretes, the portlandite tiles are positioned perpendicularly to the aggregate surface. There are fewer grains of cement at the aggregate grains surface, due to the difficulty in its packing, which leads to a local increase of w/c ratio. As a consequence, there is less cement there that can be hydrated and fill free spaces. This is why the contact zone usually has a higher porosity than the distant cement paste [31]. In the contact zone between the untreated recycled aggregate and new cement slurry the portlandite tiles and C–S–H phase are also visible (Figure 21). Figure 22 shows a fragment of the recycled aggregate after heat and mechanical treatment. On its surface there is an old cement mortar rich in durable C–S–H phases, as well as partially dehydrated calcium silicates and partial decayed products of calcite. Figure 23 shows the recycled aggregate after the heat and mechanical treatment, embedded in new concrete. The contact zone between the recycled aggregate grain and the new paste has a compact structure, which may result from strong bonding of high-quality recycling mortar containing C–S–H phases with the components of new paste, as well as the developed structure of the gravel aggregate due to applied heat and mechanical treatment. As it was noted, the rough surface of the aggregate deteriorates the portlandite orientation. Orientation of CH tiles is additionally disturbed by the presence of Ca(OH)_2_ fragments, which, being the nuclei of crystallization of this phase, causes the growth of calcium hydroxide crystals in various directions, improving mortar strength [32].

### 6.2. X-Ray Diffraction

During the proposed heat and mechanical treatment of concrete rubble, in addition to high-quality recycled aggregates, a cement mortar was also obtained, which due to the induced pozzolana properties as a result of thermal treatment can be used as a cement substitute in cement composites or as a pozzolana additive [19,20,33,34].

**Figure 24:**
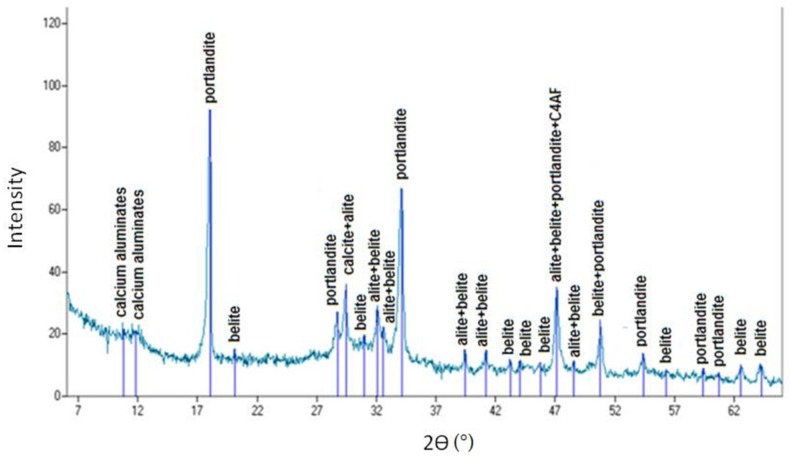
XRD patterns of cement paste from recycled aggregates without thermo-mechanical treatment.

**Figure 25:**
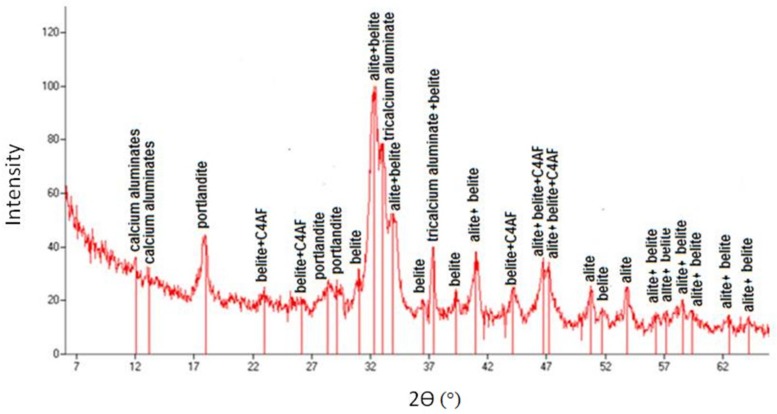
XRD patterns of cement paste from recycled aggregates after thermo-mechanical treatment (calcinating in 650 °C by 60 min).

In order to determine the effect of heat and mechanical treatment on the phase composition of cement slurry separated from the aggregate (experiment p. 6.1), the X-ray diffraction tests were realized. The samples were analysed by X-ray diffraction analysis (XRD) using a VEB FreibergerPragisionsmechanik TUR-M-62- with DRONEK radiation. All diffraction patterns were obtained by scanning the goniometer from 6 to 66 (2θ) at a rate of 0.05 min^−1^. The X-rays for unroasted and treated paste are collected, respectively, in Figure 24 and Figure 25. The scanning of the goniometer (2θ) and the absolute intensity of the reflections (*Iwz*) are also represented.

For both cement slurries the portlandite, calcium aluminates, belite, alite, as well as aluminate and aluminoferrite phases were identified. As expected, in case of material not subjected to the roasting process (Figure 24), the peak associated with the presence of portlandite was very intense, while in the sample subjected to heat treatment it was a lot less intense, which indicates a well-selected treatment temperature, allowing for almost complete decomposition of Ca(OH)_2_. The small peak from portlandite in the sample subjected to roasting (Figure 25) can be explained by high hygroscopicity of disintegrated cement slurry. The roasted sample revealed higher and more frequent peaks indicating the presence of Portland clinker components (C_3_S, C_2_S, C_3_A, C_4_AF), which are responsible for reactivity with water and for the hydration process. This explains the usefulness of roasted recycling slurry as a pozzolana additive and active filler [19,20]. The gravel aggregate improved with high-quality mortar rich in the abovementioned components enabling further hydration, which more easily reacts with new cement slurry, and which may directly translate into improved properties of cement composites. This is confirmed by the observations of other authors, who have noticed that within the recycling aggregate there is the non-hydrated cement, calcium hydroxide (CH), and dicalcium silicate (C_2_S), which are capable of hydration and creation of rehydration products [35,36,37,38].

## 7. Conclusions

According to the results of the research carried out, an appropriate heat and mechanical treatment of concrete rubble favours obtaining the coarse aggregate from high-quality recycling, of parameters similar to the natural aggregate. As a result, it can be used as a component of full value for cement concretes. The experiment revealed that when 60% of coarse natural aggregate (granite) is replaced by recycled aggregate, it is possible to obtain concrete class C55/67, i.e., a class higher than the control concrete. For comparison, using the recycled aggregate without the heat and mechanical treatment decreased the compressive strength of the concrete by three classes compared to the control concrete. In the case of other tested properties of concrete, it was observed that after roasting debris in 300–600 °C it was possible to obtain the best results, the closest to the control concrete, and sometimes even slightly better, which took place, for example, in the case of flexural strength. After the treatment, trace amounts of mortar remained on the aggregate surface, creating permanent bonds with the new cement paste and improving the investigated feature. 

Properly selected temperature of concrete rubble roasting determines the degree of aggregate cleaning, which is measured by the amount of cement mortar removed from the surface of the grains. Excessive temperature (900 °C) additionally caused damage of the granite grains, which was the main reason for a significant deterioration of aggregate properties, concrete mix consistence and the properties of hardened concrete. 

The most advantageous roasting temperature was 600 °C, which also allows to reuse the fine fraction (<4 mm). That fraction may constitute even up to 60% of concrete rubble mass. Usually such material is treated as a waste and is used for storing or filling hollows. After the heat and mechanical treatment, this fraction, rich in cement paste, partially regains its binding properties and may be successfully used as a cement substitute or as a pozzolana additive for cement composites. Moreover, a high temperature is favourable for burning the majority of impurities present in concrete rubble. Therefore, it can be stated that the proposed method of heat and mechanical treatment of concrete rubble at a temperature of 600 °C, despite the related costs, connected among other things with the necessity of building the technological line and energy costs, allows for complex use of concrete rubble for construction purposes, thus replacing non-renewable materials. Therefore, it is a highly ecological solution, in line with the idea of sustainable development of the world and requires further research in this direction.

## 8. Patent

Based on the results of research on aggregate and recycling mortar obtained according to the proposed heat and mechanical treatment of concrete rubble, a prototype of a device for comprehensive recycling of concrete rubble was designed. The device in its design is similar to the rotary furnace, equipped with two chambers: one for roasting and another one for abrasion and crushing. As a result of experimentally selected processing parameters, it is possible to obtain high-quality recycling aggregate and recycling mortar of pozzolana properties. The process of separating the mortar from the coarse aggregate and its partial disintegration is realized in one device. The concrete rubble first reaches the roasting chamber, where it is simultaneously heated to 600–650 °C, assuring almost complete dehydration of cement in the mortar and cyclic lifting on four shelves embedded in equal spaces on a rotating device. As a result, the debris heats up evenly and quickly, and collides with the machine jacket and shelves when falling. Therefore, the mortar is initially separated from the aggregate. Then the material is gradually moved towards the grinding and crushing chamber, where the mortar is finally separated from the aggregate and partially crushed by passing through a system of three rollers. Then the cement mortar together with cleaned recycling aggregate goes outside the machine, e.g., onto the bucket conveyor. The device, as well as the way the concrete rubble is processed, has become the subject of patent PAT.229887 called “Method of separating the hardened cement mortar from the coarse aggregate and disintegrating this mortar, and the device for using this method” [39].

## Figures and Tables

**Table 1:** The composition of the concrete mixture on 1 m^3^.

Component	Content
Sulphate Resistant Cement CEM I 42.5 N HSR/NA, kg	360
Sand 0–2 mm, kg	641
Granite 2–8 mm, kg	450
Granite 8–16 mm, kg	720
Plasticizer, dm^3^	3.2
Superplasticizer, dm^3^	2.48
Air-entraining admixtures, dm^3^	2.98
Water, dm^3^	144

**Table 2:** Average test results of compressive and flexural strength of concrete samples.

Average Compressive Strength (MPa)	Standard Deviation	Concrete Class	Average Flexural Strength (MPa)	Standard Deviation
67.0	8.56	C 35/45	7.61	0.58

**Table 3:** The amount of separated cement mortar.

Temperature (°C)	Mechanical Treatment Time (min)	The Amount of Separated Cement Mortar (mass %)
300	5	24
300	10	32
300	15	36
600	5	35
600	10	38
600	15	42
900	5	81
900	10	95
900	15	96

**Table 4:** Variables in the experiment plan.

X_1_	Temperature of Recycled Aggregate Heat Treatment	300	600	900	°C
X_2_	Mechanical treatment time	5	10	15	min
X_3_	Amount of recycled aggregate fraction 4–16 mm	20	40	60	%

**Table 5:** Sequential Hartley’s PS/DS-P:Ha3 plan.

Series	Real Variables			Coded Variables		
	X_1_ (°C)	X_2_ (min)	X_3_ (%)	x_1_	x_2_	x_3_
1	300	15	40	−1	1	0
2	600	10	20	0	0	−1
3	900	15	20	1	1	−1
4	300	5	60	−1	−1	1
5	900	15	60	1	1	1
6	600	5	40	0	−1	0
7	300	15	60	−1	1	1
8	300	5	20	−1	−1	−1
9	900	5	60	1	−1	1
10	900	5	20	1	−1	−1
11	300	10	40	−1	0	0
12	300	15	20	−1	1	−1
13	Control with RCAwt		60	-	-	-
14	Control with NA (granite)			-	-	-

Where: RCAwt—recycled concrete aggregate without treatment; NA—natural aggregate. Testing of concrete properties was carried out after 28 days of curing of the samples in laboratory conditions.

**Table 6:** The composition of the concrete mixes on 1 m^3^ depending on the content of recycled aggregates.

Component	Unit	Concrete Mix Composition According to Different Amount of RCA (%)			
		0%	20%	40%	60%
Cement CEM I 42.5R	kg/m^3^	400	400	400	400
w/c	-	0.40	0.40	0.40	0.40
Water	dm^3^/m^3^	158.0	158.0	158.0	158.0
Superplasticizer	dm^3^/m^3^	2.0	2.0	2.0	2.0
Sand 0–2 mm	kg/m^3^	659.4	659.4	659.4	659.4
Gravel 2–4 mm	kg/m^3^	164.9	164.9	164.9	164.9
Granite4–8 mm	kg/m^3^	453.6	362.9	272.2	181.4
Granite 8–16 mm	kg/m^3^	721.6	577.3	433.0	288.7
RCA 4–8 mm	kg/m^3^	0.0	82.9	165.8	248.7
RCA 8–16 mm	kg/m^3^	0.0	131.9	263.8	395.7

After 28 days of curing physical and mechanical properties were tested.

**Table 7:** Properties of recycling and natural aggregates.

Type of Aggregate	Calcination Temperature (°C)	Bulk Density in the Dry State (g/cm^3^)	Bulk Density in the Saturated Surface Dry (g/cm^3^)	Water Absorption (%)
RCA 4–8 mm	300	1.21	1.26	3.80
RCA 8–16 mm	300	1.19	1.24	3.48
RCA 4–8 mm	600	1.26	1.29	3.11
RCA 8–16 mm	600	1.24	1.26	3.70
RCA 4–8 mm	900	1.16	1.20	6.00
RCA 8–16 mm	900	1.14	1.21	8.00
RCAwt 4–8 mm	-	0.93	1.10	6.85
RCAwt 8–16 mm	-	0.92	1.08	7.83
NA 4–8 mm	-	1.30	1.31	1.30
NA 8–16 mm	-	1.28	1.29	1.30

**Table 8:** Results of fresh concrete mixtures**.**

Series	Slump Test (mm)	Consistency Class	Density of the Mixture (kg/m^3^)
1	175	S4	2374
2	200	S4	2340
3	170	S4	2334
4	175	S4	2290
5	0	-	2281
6	185	S4	2370
7	200	S4	2356
8	180	S4	2372
9	0	-	2275
10	160	S4	2331
11	180	S4	2353
12	200	S4	2391
13	140	S3	2340
14	225	S5	2420

**Table 9:** The average results of concrete properties tests for individual experimental series and for control series.

Series	f_cm,28_ (MPa)	Concrete class	f_fm,28_ (MPa)	E (GPa)	D (kg/m^3^)	WA (%)	WP (mm)	WC (%)
1	71.50	C55/67	7.60	37.8	2318.00	4.0	50	3.08
2	68.00	C50/60	7.74	37.2	2290.00	3.9	40	3.06
3	51.00	C35/45	7.07	34.6	2259.67	4.6	50	3.20
4	57.00	C45/55	8.26	35.2	2234.67	4.5	55	3.25
5	46.75	C30/37	6.63	33.0	2231.50	4.8	65	3.68
6	70.00	C55/67	8.59	37.7	2281.00	4.0	43	2.98
7	66.25	C50/60	8.53	36.5	2284.67	4.4	45	3.10
8	73.50	C55/67	7.53	36.6	2294.67	4.2	43	3.21
9	59.25	C45/55	6.82	36.4	2224.67	5.0	63	3.97
10	56.50	C45/55	6.88	35.2	2279.67	4.5	48	3.00
11	67.50	C50/60	7.91	36.9	2282.67	4.1	52	3.48
12	59.00	C40/50	7.17	37.0	2299.00	4.2	43	3.15
13	55.25	C35/45	6.79	34.0	2249.33	5.6	65	4.19
14	60.50	C50/60	5.98	38.5	2313.33	3.7	48	3.11

**Table 10:** The equations list for each properties of concrete.

Properties	Equations	R^2^
Compressive strength, f_cm_, MPa	= 70.2 − 4.42 × x_1_ − 2.48 × x_3_ − 6.03 × x_1_^2^ − 5.76 × x_3_^2^ − 2.86 × x_1_x_2_ + 3.09 × x_2_x_3_	0.70
Flexural strength, f_fm_, MPa	= 8.21 − 0.49 × x_1_ + 0.21 × x_3_ − 0.92 × x_1_^2^ − 0.32 × x_1_ × x_3_	0.69
Young’s modules, E, GPa	= 37.54 − 0.75 × x1 − 0.29 × x_3_ − 1.24 × x_1_^2^ − 0.74 × x_1_ × x_2_	0.70
Volume density, D, kg/m^3^	= 2281 – 16.4 × x_1_ − 21.4 × x_3_ − 10.6 × x_1_x_2_	0.68
Water absorption, WA, %	= 3.87 + 0.20 × x_1_ + 0.17 × x_3_ + 0.41 × x_1_^2^	0.71
Water permeability, WP, mm	= 47.21 + 4.85 × x_1_ + 5.48 × x_3_ + 10.31 × x_1_^2^	0.84
Water capillarity, C, %	-	-
